# Glial Contributions to Lafora Disease: A Systematic Review

**DOI:** 10.3390/biomedicines10123103

**Published:** 2022-12-01

**Authors:** Stefania Della Vecchia, Maria Marchese, Filippo Maria Santorelli

**Affiliations:** 1 Molecular Medicine and Neurogenetics, IRCCS Stella Maris Foundation, Calambrone, 56128 Pisa, Italy; 2 Neurobiology, IRCCS Stella Maris Foundation, Calambrone, 56128 Pisa, Italy

**Keywords:** Lafora disease, astrocytes, microglia, neuroinflammation, neurodegeneration, epilepsy

## Abstract

Background: Lafora disease (LD) is a neurodegenerative condition characterized by the accumulation of polyglucosan bodies (PBs) throughout the brain. Alongside metabolic and molecular alterations, neuroinflammation has emerged as another key histopathological feature of LD. Methods: To investigate the role of astrocytes and microglia in LD, we performed a systematic review according to the PRISMA statement. PubMed, Scopus, and Web-of-Science database searches were performed independently by two reviewers. Results: Thirty-five studies analyzing the relationship of astrocytes and microglia with LD and/or the effects of anti-inflammatory treatments in LD animal models were identified and included in the review. Although LD has long been dominated by a neuronocentric view, a growing body of evidence suggests a role of glial cells in the disease, starting with the finding that these cells accumulate PBs. We discuss the potential meaning of glial PB accumulations, the likely factors activating glial cells, and the possible contribution of glial cells to LD neurodegeneration and epilepsy. Conclusions: Given the evidence for the role of neuroinflammation in LD, future studies should consider glial cells as a potential therapeutic target for modifying/delaying LD progression; however, it should be kept in mind that these cells can potentially assume multiple reactive phenotypes, which could influence the therapeutic response.

## 1. Introduction

Lafora disease (LD) is a severe neurodegenerative condition belonging to the group of progressive myoclonic epilepsies (PME). It is a rare genetic disorder linked to mutations in the *EPM2A* [1] and *EPM2B* genes [2], which respectively encode laforin and malin, components of a functional complex [3]. LD’s histopathological hallmark is the accumulation of polyglucosan bodies (PBs) scattered throughout the brain [4,5]. Despite numerous advances, the pathogenesis of LD remains unclear, and the functions of laforin and malin and their role in neurodegeneration are only partially understood. In addition to alterations in glycogen metabolism, animal models of LD revealed alterations in several metabolic and molecular pathways [6,7] that may influence each other and play a role in the disease [6,7]. These include, in particular, the alteration of autophagy [8,9,10,11,12,13,14] and the ubiquitin–proteasome system [15,16,17], the impairment of the heat shock response [16,18], increased oxidative stress, and mitochondrial dysfunction [19,20].

In the recent years, neuroinflammation has also emerged as a key histopathological feature in animal models of LD [14,21,22,23]. Although, neuroinflammation begins as a defensive response [24], sustained inflammatory responses that involve microglia and astrocytes are detrimental and can contribute to neurodegeneration [25,26]. Astrocytes and microglia perform numerous housekeeping functions in the brain [27]. However, these cells also show highly variable phenotypes, depending on the environmental stimuli received and the brain region in which they are located, and in certain situations they can therefore become a source of damage. Neurotoxic and protective phenotypes have been documented in each of these cell types [25,28]; this distinction, however, is now thought to be too simplistic, as both microglial and astrocytic cells can assume multiple reactive phenotypes, even within the same disease [29,30]. Proinflammatory microglia can exert their damaging effects by producing many inflammatory mediators, including tumor necrosis factor alpha (TNF-α), interleukin 1 beta (IL-1β), interleukin 6 (IL-6), nitric oxide synthase (NOS), and reactive oxygen species (ROS) [31]. Furthermore, they upregulate several lipid-metabolism-related genes, phagosome-related genes, and cathepsins [32] and downregulate genes expressed by homeostatic microglia [33]. Some authors have referred to these CNS-damaging microglia, present in many neurodegenerative diseases, as ‘disease-associated microglia’ (DAM) [34,35]. On the contrary, protective microglia produce anti-inflammatory cytokines such as interleukins 4, 10, and 13 (IL-4, IL-10, and IL-13) and transforming growth factor beta (TGF)-β); upregulate markers such as arginase-1 (ARG-1), found in inflammatory zone 1 (FIZZ-1), CD206 pattern recognition receptor, also known as mannose receptor C type 1, and chitinase 3-like 3 (YM-1) phagocytosis genes [32]; and promote repair mechanisms [31]. Similar to these microglia, proinflammatory reactive astrocytes lose their homeostatic functions, in this case upregulating genes such as glial fibrillary acidic protein (GFAP) and complement cascade genes and releasing proinflammatory factors (e.g., IL-1β, TNF-α, and NO), damaging nervous tissue [28,36]. In contrast, neuroprotective astrocytes upregulate many neurotrophic factors and thrombospondins [36] and can release anti-inflammatory cytokines such as IL-4, IL-10, and TGF-β [37].

In this review we systematically examined studies investigating the roles of glial cells in neuroinflammation in LD. Several studies have claimed a contribution of astrocytes and microglia in LD, starting with the evidence that these cells also accumulate PBs [38,39,40,41]. With these premises, we tried to understand the roles of astrocytes and microglia in LD, the significance of PB accumulations in glial cells, potential triggers that activate astrocytes and microglia, and the possible contribution of glia to neurodegeneration and to the epileptic phenotype of LD.

## 2. Materials and Methods

A systematic literature review was conducted using the Preferred Reporting Items for Systematic Reviews and Meta-Analyses (PRISMA) guidelines [42]. The PubMed, Scopus, and Web of Science databases were searched from their start date to October 2022 (12 October 2022). The reference lists of all included studies were also searched for additional relevant citations. The authors used the following search strategy: (Lafora disease) AND ((glia) OR (microglia) OR (astrocyt*) OR (gliosis) OR (cytokin*) OR (inflammat*)). The search included only original studies. We used the research tool “Zotero” (https://www.zotero.org/download/, accessed on 12 October 2022) to collect all the results in a single library. Abstracts were retrieved using our search strategy, and duplicates were removed. The full text of all potentially eligible articles and their Supplementary Information were obtained and independently assessed by two authors (S.D.V. and M.M.). We resolved any ambiguities about eligibility through discussion. Studies were included if they reported information on (1) astrocytes, microglia, and/or neuroinflammation in LD or (2) the effects of therapeutic strategies on astrocytes, microglia, and/or neuroinflammation in LD. The exclusion criteria were as follows: (a) duplicates; (b) studies that did not relate to the objective of the article; (c) articles written in a language other than English or Italian; (d) reviews and meeting and workshop abstracts; and (e) books. The data were extracted manually by two authors and were summarized in tables. Extrapolated data concerned the neuropathological features (the presence or absence of LBs, microgliosis, astrogliosis, and inflammatory cytokines) and the phenotypical features (the presence or absence of epileptic manifestations, motor impairment, and cognitive impairment) of animal models of LD. Whenever possible, the same data were analyzed at various developmental stages and after any therapeutic interventions. We extracted data on the type of treatment (the inhibition of glycogen synthesis or alternative strategies), the start and duration of treatment, the treatment effect on neuropathological features (LBs and neuroinflammation), and clinical features (epileptic manifestations, motor impairment, and cognitive impairment). Data on the start and duration of treatment were described as continuous variables, and those on the treatment effect were described as categorical variables (neuropathological features: no effect, rescue, reduction, or increase; epileptic manifestations: no effect, rescue, reduction, or increase; and motor and cognitive impairment: no effect, improved, or worsened). The studies were grouped in tables according to the main topics covered: the presence or absence of neuroinflammation at different stages of the disease and the neuropathological and clinical effects of treatments inhibiting glycogen synthesis or alternative strategies. For the narrative review, the following main topic paragraphs were identified: PB accumulation in glial cells, neuroinflammation in animal models of LD, glial cell activation factors in LD, and the contribution of glial cells to neurodegeneration and the epileptic phenotype of the disease. The PRISMA checklist can be found in Supplementary Material. The systematic review protocol was registered on PROSPERO with the following code: 367094.

## 3. Results

### 3.1. Database Search Process

**Figure 1:**
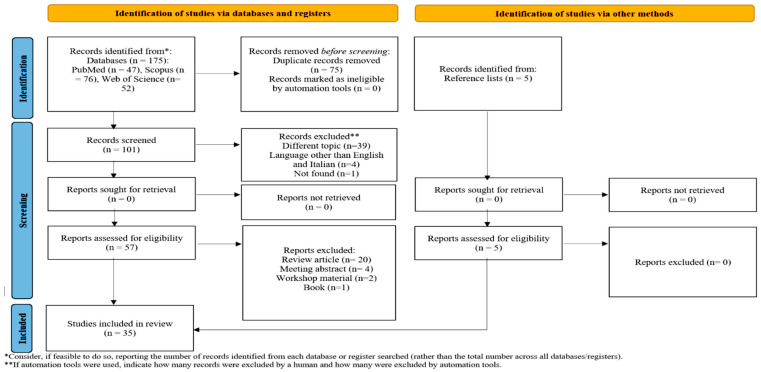
PRISMA flow chart of the review process.

The PRISMA flow chart of the review process is presented in Figure 1. The search on PubMed, Scopus, and Web of Science provided a total of 176 records. After the correction of duplicates, 101 records remained. Of these, 39 were excluded because they dealt with a topic different than the topic of interest, 4 were excluded because they were written in a language other than English or Italian, a single manuscript could not be found, 26 were excluded because they were reviews, meeting abstracts, or editorial comments, and 1 was excluded because it was a book. Five additional studies were selected by checking the references of the identified relevant papers. A total of 35 studies were then identified for inclusion in the current review [10,12,14,18,21,22,23,38,39,40,41,43,44,45,46,47,48,49,50,51,52,53,54,55,56,57,58,59,60,61,62,63,64,65,66].

### 3.2. Identification of PBs in Glial Cells

The PBs found in LD have long been assumed to accumulate only in neurons [10], which seems paradoxical given that glycogen metabolism in the brain occurs mainly in glial cells [67]. In fact, *postmortem* histopathological studies on the brains of individuals [45,48] and dogs with LD [55,57] have shown the presence of PBs in both neurons and glial cells, including astrocytes [48], as well as some PBs in extracellular locations [48]. Using a malin-deficient mouse model (*Epm2b*^−/−^), Valles-Ortega and colleagues [41] showed the presence of PBs in both astrocytes and neurons and that astrocytic PBs form earlier than the neuronal ones. However, while neurons degenerate as they accumulate PBs, astrocytes remain viable, suggesting that neurons are more sensitive than astrocytes to cell death induced by glycogen overload [68]. A subsequent study performed in *Epm2a*^−/−^ and *Epm2b*^−/−^ mice confirmed that PBs colocalize in neurons, astrocytes, and microglial cells [40] and that the formation of astrocytic PBs precedes that of neuronal PBs, as is the case of other glycogen storage diseases [69]. Interestingly, the PBs that accumulate in astrocytes are different from those found in neurons [38]. Research in malin KO mice has identified corpora-amylacea-like bodies (CALs), which are different from the neuronal Lafora bodies (nLBs) found in astrocytes; however, glycogen synthesis is required for the formation of both [70]. These two types of PBs differ in both location (astrocytic CALs are found predominantly in the hippocampus, while nLBs are mainly in the cerebral cortex) and constitution [38]. In particular, CALs, unlike nLBs, contain neo-epitopes recognized by natural IgM [38], which suggests that they may be eliminated by mechanisms of natural immunity, in particular by macrophages, after being extruded from the CNS or in the CNS itself in the case of blood–brain-barrier disruption [71]. The protein p62, which plays a role in secretory autophagy, seems to be involved in this elimination process [38]. Furthermore, this protein also seems to be important for PB formation; in fact, its deletion in malin KO mice altered the morphology of brain PBs, making them more toxic and worsening neuroinflammation and susceptibility to kainate-induced epilepsy [39].

### 3.3. Neuroinflammation as a New Hallmark of LD

Alongside the accumulation of PBs in both neuronal and glial cells, neuroinflammation has emerged as another key histopathological feature in LD animal models [21,22]. It appears in the very early stages and worsens over time, which suggests a role in the progression of the disease [21,22]. Most of the available studies on this topic have been conducted in mouse models and investigated neuroinflammation through immunostaining studies, in order to investigate astrogliosis and microgliosis, and through the analysis of mRNA and protein levels of inflammatory mediators [21,22]. A transcriptomic analysis on the brains of laforin- and malin-deficient mice at different ages (3 to 16 months) revealed that most LD mouse models already show reactive astrogliosis and microgliosis at around 3 months of age, together with the upregulation of inflammatory mediators belonging mainly to the class of inflammatory chemokines, such as ccl4, ccl5, and cxcl10. Over time, a progressive upregulation of genes involved in immune and inflammatory responses occurs, 60% of which encode proteins specific to microglia and 26% of which encode proteins shared by microglia and other cell types (astrocytes and endothelial cells) [21]. In particular, there is an upregulation of inflammatory cytokines (TNF-a, IL-1β, and IL-1a) [18,22,39,62,63,66]; complement factors (C1q, C1ql1, C3, C3ar1, and C4b) [22,39,51,56]; chemokines (cxcl10, ccl2, ccl3, ccl4, ccl5, ccl12, etc.) [21,22,39,44,51,56,63]; other mediators of immune responses, such as hepatic lipocalin-2 (Lcn2) [21,44,51,56]; cyclooxygenage-2 (COX-2)-producing prostaglandins [18,22,62]; and iNOS-producing free radicals [39]. All these mediators could contribute to neurodegeneration [72]. Table 1 summarizes the existing data on neuroinflammation in LD mouse models.

Our analysis of data from transcriptomic [21] and PCR analyses [18,22,39,62,63,66] performed in mouse models of LD revealed the upregulation of several genes characteristically expressed in proinflammatory microglia [33,73,74], such as lipid-metabolism- and phagosome-related genes (Cst7, Lyz2, Clec7a, Axl, and Itgax), cathepsins (Ctss and Ctsz), major histocompatibility complex (MHC-II)-related genes (H2-D1 and H2-K1), proinflammatory genes (Tlr2, Il1b, and TNFa), and Trem2, a receptor required for DAM activation [75]. One of these studies [39], performed in a 12-month-old Epm2b KO mouse model, also showed the upregulation of several anti-inflammatory genes such as IL-10, IL-10ra, TGF-β, S100A-10, IL-13, IL-4, ARG-1, and CD206, which are characteristic of glia polarized towards a protective phenotype. ARG-1 and IL10ra upregulation was also found in an RNA-Seq analysis performed on *Epm2a*^−/−^ and *Epm2b*^−/−^ mice at 16 months of age [21]. Another study, instead, did not confirm the upregulation of Arg-1 in a malin KO mouse model [63].

The presence of astrogliosis and microgliosis was also confirmed by histopathological studies on brain sections from dogs with LD [49,50].

The early activation of glial cells has also been observed in a laforin-deficient zebrafish model [14], while the upregulation of *gfap* also confirmed astrocyte involvement in *epm2a^−/−^* zebrafish larvae [14]. Furthermore, the analysis of inflammatory and glia-specific genes suggests that differently polarized glial populations may coexist in the zebrafish LD model. The upregulation of *tnfa*, *cox2b,* and *il1b* and genes such as *csfr1a* suggests the presence of proinflammatory activated cells [76,77]; in the case of *csfr1a*, in particular, this upregulation may, through a positive feedback mechanism, help to support the DAM signature by interacting with trem2 signaling [76]. Finally, the upregulation of il10 suggests that at least some microglial cells may also be activated in an anti-inflammatory manner in the LD zebrafish model [78].

### 3.4. What Triggers Glial Cell Activation?

The question of what triggers the activation of glial cells in LD is still debated. It is very likely that multiple factors (neuronal death, polyglucosan accumulation, altered autophagy, etc.) play a part, especially considering that these are very active cells that are capable of responding to minimal changes in their environment [79,80].

The inhibition of glycogen synthesis has been shown to have positive effects on neuroinflammation, suggesting a possible role of PB accumulation in glial cell activation. Table 2 summarizes the neuropathological and clinical effects of glycogen synthesis inhibition in several mouse models of LD [10,43,44,51,56,58,59,60,63,64]. Interestingly, the inhibition of astrocytic glycogen synthesis appears to be sufficient to rescue astrogliosis and microgliosis as well as the impairment of autophagy and metabolic alterations observed in LD mice [51]. These data suggest that astrocytic PBs may be responsible for the activation of both astrocytes and microglia. However, to date there are no studies targeting microglial PBs.

Positive effects on neuroinflammation were also obtained using drugs with anti-inflammatory actions or acting on other molecular/metabolic pathways found to be impaired in LD [18,23,62]. Table 3 summarizes the neuropathological and clinical effects of the administration of these drugs in animal models of LD. These data seem to confirm the hypothesis that different factors may contribute to the development of neuroinflammation. Autophagy stimulators [46,62], chaperones [46], antioxidants [46,61], and drugs that increase the heat shock response [18], often without any action on PBs, have also been shown to reduce or rescue neuroinflammation in LD murine models. In many cases, however, it is difficult to determine how much the reduction in neuroinflammation depends on a direct anti-inflammatory action or a specific activation of chaperone pathways. For example, dexamethasone has a known anti-inflammatory action but also acts by increasing the heat shock response [18]. Moreover, metformin, which promotes autophagy through the activation of AMP-activated protein kinase (AMPK) and acts as a neuroprotective agent in several neurodegenerative diseases [81], and 4-PBA, a chemical chaperone that sequesters misfolded and aggregated proteins associated with several human neurodegenerative diseases [82], also reduced PBs and the susceptibility to induced seizures [46,47]. Cannabidiol, on the other hand, had no effect on neuroinflammation or seizure susceptibility but reduced cognitive impairment in a mouse model of LD [66].

Interesting results emerged from the larval zebrafish model of LD [14]. Up to 5-day-old laforin-deficient zebrafish larvae do not develop PBs but show several metabolic/histopathological alterations, including altered autophagy and neuroinflammation, and are already epileptic, suggesting a role of alternative factors to PBs in neuroinflammation in the epileptic phenotype, at least in zebrafish [14].

### 3.5. Contribution of Glial Cells to Neuronal Dysfunction and Death

Albeit variable in degree, LD mice exhibit neurodegeneration with neuronal loss and the activation of astrocytes and microglia [10,40,41,46,83]. However, the mechanisms underlying the neuronal death in LD remain unknown. Some studies in mouse models of LD support a role for PBs [68,84,85,86]. On the contrary, an increase in PB-independent apoptosis death was observed in the laforin-deficient zebrafish model [14]. A form of cell death called ‘dark cell death’, which precedes the formation of PBs in neurons, has also been described in some mouse models of LD [83,87]. Many degenerated neurons furthermore do not show visible PBs [83,87]. Taken together, these data suggest that different mechanisms of neuronal death may coexist in LD. As suggested by a recent study showing that neurodegeneration may be linked to the accumulation of PBs in astrocytes [51], glial cells could also play a role. In particular, astrocytic PBs appear to be responsible for proinflammatory glial cell activation, triggering a persistent inflammatory reaction [51]. Proinflammatory reactive glial cells could participate in neuronal death both because they lose important homeostatic functions and because they gain neurotoxic properties [28,88,89].

One of the most important homeostatic roles of astrocytes is the clearance of the K^+^ and glutamate released during neuronal activity, which they perform through the Kir4.1 channel and the EAAT2 transporter [90,91]. If these processes are impaired, neuronal excitability increases to the point of excitotoxicity, leading to cell death [92,93]. Indeed, different mechanisms may impair K+ and glutamate clearance by astrocytes in LD. One is the accumulation of glycogen in astrocytes, given that these processes depend on glycogenolysis [38,40,69,94,95]. Second, inflammatory cytokines (e.g., TNFα and IL-6) released by proinflammatory glial cells reduce glutamate uptake by EAAT2 transporters and promote its astrocytic release [96,97,98]. A third mechanism demonstrated in cellular [52] and mouse models [53] of LD is the functional reduction in the astrocytic EEAT2 transporter [52,53] linked to its altered ubiquitination and recycling due to the deficiency of the laforin/malin complex [54].

Another important glial cell function is the trophic support provided to neurons through the release of several growth factors (e.g., BDNF and NGF) [36,88,99]. Proinflammatory reactive glial cells stop releasing neurotrophins [36,88], and even though reduced levels of neurotrophins (e.g., BDNF and NGF) were demonstrated in the cerebral cortex of a laforin-deficient mouse model [100], the possibility that proinflammatory glia contribute to neuronal death in this way cannot be ruled out [36,88].

Alongside this loss of their homeostatic functions, glial cells activated in a proinflammatory sense may acquire neurotoxic properties [101]. Proinflammatory glial cells release greater amounts of free radicals and conversely produce fewer antioxidant agents [102,103,104], thereby contributing to the increased oxidative stress and the reduction in detoxifying enzymes observed in mouse models of LD [20,105]. Proinflammatory astrocytes and microglia also release a multitude of mediators that are toxic to neurons. Ccl4 and Ccl5 are mainly involved in the infiltration and activation of immune cells [106], including microglia [107], and have been implicated in the progression of several neurodegenerative diseases [108,109]. Cxcl10, produced predominantly by astrocytes, recruits microglia [110] and, by binding to chemokine, CXC motif, receptor 3 on neurons, can induce neuronal dysfunction and death [111,112]. Lcn2, secreted by proinflammatory astrocytes in a mouse model of LD, is selectively toxic to neurons but harmless to glial cells [113]. In general, the increase in chemokines observed in LD animal models (Cxcl10, Ccl2, Ccl4, Ccl5, etc.) may favor the infiltration of peripheral immune cells such as monocytes into the brain and promote the persistence of neuroinflammation [114]. As in other neurodegenerative diseases [115], it is possible that in LD the increased expression of local chemokines induces the infiltration of peripheral immune cells that contribute to the persistence if neuroinflammation.

Proinflammatory cytokines (e.g., IL6, IL1B, and TNFa) released by both proinflammatory microglia and astrocytes [101] can cause synaptic dysfunction, neuronal death, and the inhibition of neurogenesis [116]. In particular, it has been shown that TNF released by inflamed microglia, in conjunction with IL-1α and C1q, drives the damaging activation of astrocytes, leading to a final toxic effect on neurons in various neurodegenerative disorders [117]. Since elevated TNF- α, IL-1α, and C1q levels were also found in LD animal models, a role of this molecular pathway in the neurodegeneration of LD cannot be excluded.

The observation of the upregulation of the complement system, in particular of the components C1q [39] and C3 factors [39,44,51,56], in mouse models of LD suggests that the inappropriate phagocytosis of synapses and neurons by reactive astrocytes and microglia may contribute to the progression of LD [118]. C1q produced mainly by reactive microglia and C3 produced by reactive astrocytes [119] induce neuronal dysfunction and phagocytosis [76,119,120,121]. Trem2 receptors, which are expressed by reactive microglia and are upregulated in mouse models of LD [21,39], may also be involved in in the phagocytic process [76].

COX-2, increased in several models of LD, contributes to neuroinflammation and possibly neurodegeneration through the production of prostanoids [122]. Although positive effects of a COX-2 inhibitor, ibuprofen, were recently described in a mouse model of LD [123], a high dose of ibuprofen was used, and in humans, chronic treatment with high doses of ibuprofen is associated with health risks [123]. However, it might be worth testing drugs that selectively inhibit COX-2 and are associated with fewer systemic side effects.

It is also important to remember that immune cells, including microglia, play an important role in the regulation and distribution of brain iron. In addition, inflammatory cytokines such as TNF-α and IL-6 induce the expression of iron transport receptors and promote iron accumulation in neurons and microglia, which may contribute to neuroinflammation [124]. Therefore, it would be of interest to test the possible accumulation of iron in LD animal models.

Data from the studies of the mouse and zebrafish models of LD included in this review suggest that a portion of glial cells may exhibit an anti-inflammatory phenotype that, contrary to what has been seen so far, could have a neuroprotective effect. To date, however, this hypothesis has not been fully investigated.

### 3.6. Glial Contribution to LD Epileptic Phenotype

The mechanisms underlying LD epilepsy remain unknown. Since, over time, several authors have argued that it may have a multifactorial origin, in this review we focus on the possible contribution of glial cells to epileptogenesis in LD.

In animal models of LD, all drugs [18,47,61,62] and therapeutic strategies (glycogen synthesis inhibition) [10,59,60,64] found to reduce neuroinflammation also resulted in a reduction/rescue of the epileptic phenotype (Table 2 and Table 3), suggesting the existence of a causal relationship between seizure susceptibility and neuroinflammation, and thus a role for proinflammatory activated glial cells in the development of the LD epileptic phenotype. In support of this, cannabidiol, which did not improve neuroinflammation, had no effect on the epileptic phenotype [66]. Furthermore the elimination of astrocytic glycogen synthesis results in the rescue of neuroinflammation but not of the epileptic phenotype, which according to the authors, is related to the accumulation of PBs within neurons and not astrocytes [51].

Overall, the literature seems to support a role for neuroinflammation in LD epilepsy, be it driven by PBs or other mechanisms (impaired autophagy, oxidative stress, reduced response to thermal stress, etc.). Neuroinflammation and epilepsy are known to influence each other [125,126]. Data obtained in mouse models of LD show that neuroinflammation precedes the development of the epileptic phenotype, suggesting that activated glial cells may play a causative role in its development. However, epileptic activity may then, in turn, aggravate the inflammatory phenotype, thus establishing a positive feedback loop that amplifies the neuronal damage.

The mechanisms by which glial cells may contribute to LD epilepsy are multiple. First, impaired K^+^ and glutamate clearance, as seen above, can lead to neuronal hyperexcitability and contribute to epileptogenesis. In this regard, in addition to the aforementioned data on EAAT2 [52,53,54], we recently observed the upregulation of the astrocytic Kir4.1 channel in a new laforin-deficient zebrafish model [14]. The downregulation of this channel has generally been described in inflammatory states, but impaired function of Kir4.1 has also been associated with autism–epilepsy phenotypes in humans [127], and we therefore cannot exclude a possible role for it in the epileptic phenotype observed in epm2a KO larvae. Second, proinflammatory glial cells produce numerous inflammatory mediators that may contribute to the development of epileptic activity [125]. Many of the inflammatory signaling pathways that have been implicated in epileptogenesis are upregulated in animal models of LD and could be effective targets for seizure treatment. Third, proinflammatory glial cells may also contribute to LD epileptogenesis by increasing the production of oxygen and nitrogen radicals [128,129]. Indeed, in mouse models of LD, increased oxidative stress [20,105] and the upregulation of iNOS [39], which reactive glial cells can produce in large quantities, were observed.

## 4. Discussion

This paper systematically reviews published data dealing specifically with the role of astrocytes and microglia in neuroinflammation in LD. Although LD has long been approached from a “neuronocentric” perspective, there is growing evidence suggesting that glial cells also play a part in its disease mechanisms, in line with what is seen in most neurological [29,130] and neurodegenerative conditions [131].

The first important observation emerging from this data review concerns the discovery of PBs in glial cells [38,39,40,41,51], although the significance of glycogen accumulations in non-neuronal cells remains to be established. In particular, the literature data only allow speculation on astrocytic PBs, as microglial PBs have not yet been studied in detail. However, studies exploring the inhibition of glycogen synthesis support a pathogenic and detrimental role for astrocytic PBs in neuroinflammation, autophagic alterations, and metabolic changes [51]. The accumulation of astrocytic PBs seems to be capable, on its own, of triggering astrocyte and microglia activation [51]. Proinflammatory activated glial cells secrete several inflammatory mediators and upregulate genes typically involved in glial-damaging phenotypes. Therefore, glial cells, by losing their homeostatic functions, may contribute to the neurodegeneration and epilepsy occurring in LD. On the other hand, the observation—in line with what occurs in normal aging and in other neurodegenerative diseases [132]—that astrocytes in LD accumulate CALs [38,39,51] (also termed “wasteosomes”) [132,133], supports the suggestion that they may be waste containers that are produced to clean up the brain [132,133]. It is thought that they may be secreted by the cells [38,71] and eliminated by macrophagic phagocytosis once they reach the cervical lymph nodes through the meningeal lymphatic system [134]. On this basis, it seems possible that the difference in mortality between astrocytes and neurons accumulating PBs in LD may be due to a difference in toxicity between astrocytic CALs and neuronal PBs. However, a protective role for neuronal PBs was also suggested by observing that alterations of PB morphology, such as that caused by the deletion of p62, increase the toxicity of these cells and thus the susceptibility to epilepsy [39].

The drivers of the proinflammatory polarization of glial cells are only partially known and are potentially numerous. Whereas the accumulation of PBs in astrocytes has been shown to activate both astrocytes and microglia in a proinflammatory manner, pharmacological studies in LD mouse models have suggested that neuroinflammation may also depend on other pathways, such as impaired autophagy [62], increased oxidative stress [61], and a reduced heat stress response [18]. Similar results have also been obtained in a new zebrafish model of LD [14]. It should be noted that zebrafish have no star-shaped astrocytes, only radial glia [135] whose functions have yet to be precisely defined. However, they seem to perform several functions of classical mammalian glia, such as neurogenesis functions and homeostatic roles in neural circuits and brain barriers [135]. Therefore, the possible roles of glial cells in LD and neurodegenerative diseases in general, as well as the differences in the roles of these cells between teleost fish and mammals, remain to be elucidated.

In short, the role of microglia in LD is still underexplored, and inferences regarding this type of cell in LD are limited by the lack of targeted studies. Although PBs have also been observed in this cell type, we do not know whether they accumulate glycogen autonomously or whether they phagocytize it from outside. Certainly, characterizing microglial glycogen inclusions (e.g., CALs, LBs, or other forms) could help to clarify some issues. The finding of mediators and markers of anti-inflammatory pathways in mouse LD models [21,39] suggests that at least some microglial cells may be engaged in protective functions, for example, to eliminate cells containing PBs. The observation of *hexb* gene upregulation in a zebrafish model of LD [14] suggests that the dysregulation of lysosomal function and autophagy in microglia may contribute to the neuropathology of LD [8,9,10,11,12,13]. On the other hand, the results of analyses of inflammatory markers [18,22,39,62,63,66] and RNA-Seq analyses indicate the possible presence in LD, as in other neurodegenerative diseases, of disease-associated microglia. However, there is still a need for the targeted characterization of glial phenotypes in LD.

## 5. Conclusions

Although the recent literature is revealing knowledge gaps, and many questions remain unanswered, glial cells are clearly involved in LD. Future studies should be designed to address glial cells as a potential therapeutic target, with the aim to arrest the progression of LD. In this context, the variability in the polarization of astrocytes and microglia observed within the same disease could make treatment with anti-inflammatory drugs difficult and not entirely effective. For this reason, it is important to characterize the phenotypes of these cells in more detail and possibly combine strategies aimed at enhancing the anti-inflammatory/protective processes. Although animal models remain the ideal platforms to continue studying the disease and for drug screening, it would also be useful to study microglial and astrocytic activation in humans through fluid [136,137,138,139] and imaging biomarkers [137,140,141].

## Figures and Tables

**Table 1:** Neuroinflammation in mouse models of LD.

	16 Days	3-Month LD Mouse Models	4–5-Month LD Mouse Models	6–12-Month LD Mouse Models	16-Month LD Mouse Models
**Astrogliosis** **(**↑ **GFAP immunostaining)**	Absent in *Epm2a* KO [22] and *Epm2b* KO mice [22]	Present in *Epm2a* KO [21,22] and *Epm2b* KO mice [21,22]	Present in *Epm2a* KO [60,62] and *Epm2b* KO mice [23,46,62,63,66]	Present in *Epm2a* KO [12,18,21,22,44,58,64] and *Epm2b* KO mice [10,18,21,22,39,51,63,66]	Present in *Epm2a* KO [21,56] and *Epm2b* KO mice [21]
**Microgliosis** **(**↑ **Iba1 immunostaining)**	Absent in *Epm2a* KO [22] and *Epm2b* KO mice [22]	Present in *Epm2a* KO [22] and *Epm2b* KO mice [22]	Present in *Epm2a* KO [60,62] and *Epm2b* KO mice [23,62]	Present in *Epm2a* KO [18,22,44,58] and *Epm2b* KO mice [18,22,39,51,63,66]	Present in *Epm2a* KO [56]
**Complement system**					
**C3**	-	-	-	↑ in *Epm2a* KO mice [44,51] and *Epm2b* KO mice [39]	↑ in *Epm2a* KO mice [56]
**C4b**	-	-	-	↑ in *Epm2b* KO mice [22]	-
**C1q**	-	-	-	↑ in *Epm2b* KO mice [39]	-
**C1ql1**	-	-	-	↑ in *Epm2b* KO mice [22]	-
**C3ar1**	-	-	-	↑ in *Epm2a* KO [22] and *Epm2b* KO mice [22]	-
**Colony-stimulating factors**					
**Csf3r**	↑ in *Epm2b* KO mice [22]	-	-	↑ in *Epm2a* KO mice [22]	-
**Chemokines**					
**Ccl2**	-	-	-	↑ in laforin KO [21] and malin KO mice [21,39,51]	↑ in laforin KO [21] and malin KO mice [21]
**Ccl4**	-	↑ in laforin KO mice [22]	-	↑ in malin KO mice [22]	
**Ccl5**	-	↑ in laforin KO mice [43,65]	-	↑ in laforin KO [21,44] and malin KO mice [21]	↑ in laforin KO [21] and malin KO mice [21]
**Ccl6**	-	-	-	↑ in malin KO mice [22]	
**Ccl12**	-	-	-	↑ in laforin KO [21] and malin KO mice [21,39]	↑ in laforin KO [21] and malin KO mice [21]
**Cxcl10**	-	↑ in laforin KO [21,22,43,65] and malin KO mice [21,22,43,65]	↑ in laforin KO [21] and malin KO mice [21,23]	↑ in laforin KO [21,22,44] and malin KO mice [21,22,39,51,63]	↑ in laforin KO [21,56] and malin KO mice [21]
**Proinflammatory cytokines**					
**TNF-alpha**	↑ in laforin KO [22] and malin KO mice [22]	-	↑ in laforin KO [62] and malin KO mice [62]	↑ in laforin KO [18] and malin KO mice [18,22,39,66]	-
**TNF-gamma**	-	-	-	↑ in malin KO mice [39,66]	-
**IL6**	↑ in laforin KO [22] and malin KO mice [22]	-	-	↑ in malin KO mice [22,39,63]	-
**IL1a**	-	-	-	↑ in malin KO [39]	-
**IL1b**	-	-	↑ in malin KO mice [66]	↑ in malin KO mice [66]	-
**IL18**	-	-	-	↑ in malin KO mice [39]	-
**Anti-inflammatory cytokines**					
**TGF-beta**	-	-	-	↑ in malin KO mice [39]	-
**S100A10**	-	-	-	↑ in malin KO mice [39]	-
**IL10**	-	-	-	↑ in malin KO mice [39]	↑ in laforin KO and malin KO mice [21]
**IL10ra**	-	-	-	↑ in malin KO mice [21]	-
**IL13**	-	-	-	↑ in malin KO mice [39]	-
**IL4**	-	-	-	↑ in malin KO mice [39]	-
**ARG-1**	-	-	-	↑ in malin KO mice [39]	↑ in laforin KO and malin KO mice [21]
**CD206**	-	-	-	↑ in malin KO mice [39]	-
**Other immunological/inflammatory mediators**					
**Lcn2**	-	-	-	↑ in laforin KO [21] and malin KO mice [21,44,51]	↑ in laforin KO [21,56] and malin KO mice [21]
**Trem2**	-	-	-	↑ in malin KO mice [39]	-
**Cox-2**	-	↑ in laforin KO [22] and malin KO mice [22]	↑ in laforin KO [62] and malin KO mice [62]	↑ in laforin KO [18,22] and malin KO mice [18,22]	-
**iNOS**		-	-	↑ in malin KO mice [39]	-

↑ implies raised inflammation process.

**Table 2:** Summary of studies describing the effects of the inhibition of glycogen synthesis in animal models of LD.

Reference	Model	PBs	Neuroinflammation	Other Pathological Features	Motor, Cognitive, and Seizure/Epileptic Susceptibility Outcomes
Turnbull et al., 2011 [64]	*Epm2a* KO + R5 KO mice sacrificed at 12 mo	Massive reduction in PBs	Rescue of astrogliosis *	-	Rescue of myoclonus
Pederson et al., 2013 [59]	*Epm2a KO + Gys KO* mice sacrificed at 20–26 mo	Rescue	Rescue of astrogliosis *	Rescue of neurodegeneration	Reduced susceptibility to induced seizures
Duran et al., 2014 [10]	*Epm2b* KO + *Gys* KO mice sacrificed at 11 mo	Rescue	Rescue of astrogliosis * and microgliosis ^♦^	Rescue of autophagy impairment	Rescue of induced seizure susceptibility
Rai et al., 2017 [60]	*Epm2a* KO + leptin receptor KO mice sacrificed at 4–5 mo	Reduction	Reduction in astrogliosis * and microgliosis ^♦^	-	Reduced susceptibility to induced seizures
Israelian et al., 2020 [56]	*Epm2a* KO + R6 KO mice sacrificed at 12–15 mo	25–50% reduction in PBs	Reduction in inflammatory markers ^•^ (Lcn2 e cxcl10) but not in astrogliosis * or microgliosis ^♦^	-	No effect (nonsignificant reduction in induced seizure susceptibility)
Duran et al., 2021 [51]	*Epm2b* KO + Gys KO mice sacrificed at 3 and 11 mo	CAL rescue. nLBs still present	Rescue of astrogliosis * and microgliosis ^♦^	Rescue of autophagy impairment	No effect on induced seizure susceptibility
Gumusgoz et. al, 2021 [43]	CRISPR/Cas9-mediated disruption of Gys1 in neonatal *Epm2a* KO and *Epm2b* KO mice up to sacrifice at 3 mo	Reduction	Reduction in inflammation markers (cxcl10 and ccl5) ^•^	-	-
Ahonen et al., 2021 [44]	GYS-ASO administered to *Epm2a* KO and Epm2b KO young mice (1–2 mo) and older mice (3–8 mo)	Young mice: LB reduction. Older mice: LB reduction. The effect was stronger if treatment was earlier (3 mo vs. 8 mo), was longer (3 mo to 12 mo vs. 3 mo to 6 mo), or was dosed higher.	Older mice: reduction in astrogliosis * and inflammation markers ^•^ (Lcn2, cxcl10, C3, and ccl5) but not in microgliosis ^♦^	-	-
Nitscke et al., 2021 [58]	*Epm2a* KO + Gys KO mice tamoxifen-induced at 4 mo to sacrifice at 12 mo	Arrest of PB formation	No effect on astrogliosis * or microgliosis ^♦^	-	-
Varea et al., 2021 [63]	Malin KO + Gys KO mice tamoxifen-induced at 4 mo or 6 mo to sacrifice at 11 mo	Gys KO at 4 mo arrested PB formation. At 6 mo was less effective than at 4 mo.	No effect on astrogliosis *, microgliosis ^♦^, or inflammation markers ^•^ (IL6, Cxcl10, and CD11b)	-	-
Gumusgoz et al., 2022 [65]	Gys1-targeting AAV-amiRNA administered in neonatal Epm2a and Epm2b KO mice up to sacrifice at 3 mo	~40% reduction in PBs across the brain	Reduction in Cxcl10 level in Epm2a KO mice.	-	-

Mo: month. Gys: glycogen synthase; * GFAP immunostaining; ^♦^ Iba-1 immunostaining; ^•^ qRT-PCR. ICV: intracerebroventricular.

**Table 3:** Summary of studies describing alternative therapeutic strategies to the inhibition of glycogen synthesis in animal models of LD.

Reference	Model and Start and Duration of Treatment	PBs	Neuroinflammation	Other Pathological Features	Motor, Cognitive and Seizure/Epileptic Susceptibility Outcomes
Mollà et al., 2021 [23]	*Epm2b* KO mice + Propanolol or EGCG from 3 mo of age for 2 mo	No reduction	Reduction in astrogliosis * and microgliosis ^♦^, more effective with propanolol	Improvement in hippocampal neuronal disorganization. Close correlation between PBs and inflammation but not between PBs and neuronal defects.	Improved memory and attention
Sánchez-Elexpuru et al., 2017 [61]	*Epm2b* KO mice + Sodium Selenate from 9 mo of age for 10 weeks	No reduction	Reduction in astrogliosis *	Reduction in neuronal degeneration	Improved motor and memory deficits and reduced susceptibility to induced seizures
Sinha et al., 2021 [18]	*Epm2a* and *Epm2b* KO mice + Dexamethasone from 6 mo of age for 2 mo	No reduction	Reduction in astrogliosis *, microgliosis ^♦^, and inflammation markers ^•^ (TNF-a- and Cox-2)	Partial restoration of HSF1 levels and reduction in oxidative stress. No effect on autophagy.	Reduced susceptibility to induced seizures
Berthier et al., 2016 [46], Sánchez-Elexpuru et al., 2017 [47]	*Epm2b* KO mice + 4-PBA or metformin from 3 mo of age for 2 mo	Reduction	Reduction in astrogliosis *	Reduction in polyubiquitinated protein aggregates and neuronal loss. Enhanced expression of endogenous chaperones such as BiP/GRP78 for 4PBA.	Improved motor deficits and reduced susceptibility to induced seizures
Berthier et al., 2016 [46]	*Epm2b* KO mice + Trehalose from 3 mo of age for 2 mo	No reduction	Reduction in astrogliosis *	Enhanced expression of endogenous chaperones such as BiP/GRP78 for 4PBA.	Improved motor deficits
Sinha et al., 2021 [62]	*Epm2a* and *Epm2b* KO mice + Trehalose from 1 mo of age for 3 mo	No reduction	Reduction in astrogliosis *, microgliosis ^♦^, and inflammation markers ^•^ (TNF-a- and Cox-2)	Partial restoration of autophagy and reduction in endoplasmic reticulum stress.	Reduced susceptibility to induced seizures
Aso et al., 2020 [66]	*Epm2b* KO mice + Cannabidiol from 4 or 10 mo of age for 2 mo	Reduction in some cerebral areas	No significant effect	-	Improvement in cognitive impairment but no reduction in the susceptibility to induced seizures

Mo: month. Gys: glycogen synthase; * GFAP immunostaining; ^♦^ Iba-1 immunostaining; ^•^ qRT-PCR.

## Data Availability

Additional information on the data may be requested from the corresponding author.

## Supplementary Materials

The following supporting information can be downloaded at: https://www.mdpi.com/article/10.3390/biomedicines10123103/s1, The PRISMA checklist of the review process.

## References

[1] Minassian B.A., Lee J.R., Herbrick J.A., Huizenga J., Soder S., Mungall A.J., Dunham I., Gardner R., Fong C.Y., Carpenter S. (1998). Mutations in a gene encoding a novel protein tyrosine phosphatase cause progressive myoclonus epilepsy. Nat. Genet..

[2] Chan E.M., Young E.J., Ianzano L., Munteanu I., Zhao X., Christopoulos C.C., Avanzini G., Elia M., Ackerley C.A., Jovic N.J. (2003). Mutations in NHLRC1 cause progressive myoclonus epilepsy. Nat. Genet..

[3] Moreno D., Towler M.C., Hardie D.G., Knecht E., Sanz P. (2010). The laforin-malin complex, involved in Lafora disease, promotes the incorporation of K63-linked ubiquitin chains into AMP-activated protein kinase beta subunits. Mol. Biol. Cell.

[4] Sullivan M.A., Nitschke S., Steup M., Minassian B.A., Nitschke F. (2017). Pathogenesis of Lafora Disease: Transition of Soluble Glycogen to Insoluble Polyglucosan. Int. J. Mol. Sci..

[5] Turnbull J., Tiberia E., Striano P., Genton P., Carpenter S., Ackerley C.A., Minassian B.A. (2016). Lafora disease. Epileptic Disord..

[6] Mitra S., Gumusgoz E., Minassian B.A. (2022). Lafora disease: Current biology and therapeutic approaches. Rev. Neurol..

[7] Parihar R., Rai A., Ganesh S. (2018). Lafora disease: From genotype to phenotype. J. Genet..

[8] Aguado C., Sarkar S., Korolchuk V.I., Criado O., Vernia S., Boya P., Sanz P., de Córdoba S.R., Knecht E., Rubinsztein D.C. (2010). Laforin, the most common protein mutated in Lafora disease, regulates autophagy. Hum. Mol. Genet..

[9] Criado O., Aguado C., Gayarre J., Duran-Trio L., Garcia-Cabrero A.M., Vernia S., San Millán B., Heredia M., Romá-Mateo C., Mouron S. (2012). Lafora bodies and neurological defects in malin-deficient mice correlate with impaired autophagy. Hum. Mol. Genet..

[10] Duran J., Gruart A., García-Rocha M., Delgado-García J.M., Guinovart J.J. (2014). Glycogen accumulation underlies neurodegeneration and autophagy impairment in Lafora disease. Hum. Mol. Genet..

[11] Knecht E., Criado-García O., Aguado C., Gayarre J., Duran-Trio L., Garcia-Cabrero A.M., Vernia S., San Millán B., Heredia M., Romá-Mateo C. (2012). Malin knockout mice support a primary role of autophagy in the pathogenesis of Lafora disease. Autophagy.

[12] Puri R., Suzuki T., Yamakawa K., Ganesh S. (2012). Dysfunctions in endosomal-lysosomal and autophagy pathways underlie neuropathology in a mouse model for Lafora disease. Hum. Mol. Genet..

[13] Rao S.N., Maity R., Sharma J., Dey P., Shankar S.K., Satishchandra P., Jana N.R. (2010). Sequestration of chaperones and proteasome into Lafora bodies and proteasomal dysfunction induced by Lafora disease-associated mutations of malin. Hum. Mol. Genet..

[14] Della Vecchia S., Ogi A., Licitra R., Abramo F., Nardi G., Mero S., Landi S., Battini R., Sicca F., Ratto G.M. (2022). Trehalose Treatment in Zebrafish Model of Lafora Disease. Int. J. Mol. Sci..

[15] Garyali P., Siwach P., Singh P.K., Puri R., Mittal S., Sengupta S., Parihar R., Ganesh S. (2009). The malin-laforin complex suppresses the cellular toxicity of misfolded proteins by promoting their degradation through the ubiquitin-proteasome system. Hum. Mol. Genet..

[16] Mittal S., Dubey D., Yamakawa K., Ganesh S. (2007). Lafora disease proteins malin and laforin are recruited to aggresomes in response to proteasomal impairment. Hum. Mol. Genet..

[17] Vernia S., Rubio T., Heredia M., Rodríguez de Córdoba S., Sanz P. (2009). Increased endoplasmic reticulum stress and decreased proteasomal function in lafora disease models lacking the phosphatase laforin. PLoS ONE.

[18] Sinha P., Verma B., Ganesh S. (2021). Dexamethasone-induced activation of heat shock response ameliorates seizure susceptibility and neuroinflammation in mouse models of Lafora disease. Exp. Neurol..

[19] Lahuerta M., Aguado C., Sánchez-Martín P., Sanz P., Knecht E. (2018). Degradation of altered mitochondria by autophagy is impaired in Lafora disease. FEBS J..

[20] Romá-Mateo C., Aguado C., García-Giménez J.L., Knecht E., Sanz P., Pallardó F.V. (2015). Oxidative stress, a new hallmark in the pathophysiology of Lafora progressive myoclonus epilepsy. Free Radic. Biol. Med..

[21] Lahuerta M., Gonzalez D., Aguado C., Fathinajafabadi A., García-Giménez J.L., Moreno-Estellés M., Romá-Mateo C., Knecht E., Pallardó F.V., Sanz P. (2020). Reactive Glia-Derived Neuroinflammation: A Novel Hallmark in Lafora Progressive Myoclonus Epilepsy That Progresses with Age. Mol. Neurobiol..

[22] López-González I., Viana R., Sanz P., Ferrer I. (2017). Inflammation in Lafora Disease: Evolution with Disease Progression in Laforin and Malin Knock-out Mouse Models. Mol. Neurobiol..

[23] Mollá B., Heredia M., Sanz P. (2021). Modulators of Neuroinflammation Have a Beneficial Effect in a Lafora Disease Mouse Model. Mol. Neurobiol..

[24] Wyss-Coray T., Mucke L. (2002). Inflammation in neurodegenerative disease—A double-edged sword. Neuron.

[25] Kempuraj D., Thangavel R., Natteru P.A., Selvakumar G.P., Saeed D., Zahoor H., Zaheer S., Iyer S.S., Zaheer A. (2016). Neuroinflammation Induces Neurodegeneration. J. Neurosurg. Spine.

[26] Russo M.V., McGavern D.B. (2016). Inflammatory neuroprotection following traumatic brain injury. Science.

[27] Verkhratsky A., Parpura V., Li B., Scuderi C. (2021). Astrocytes: The Housekeepers and Guardians of the CNS. Adv. Neurobiol..

[28] Glass C.K., Saijo K., Winner B., Marchetto M.C., Gage F.H. (2010). Mechanisms underlying inflammation in neurodegeneration. Cell.

[29] Bachiller S., Jiménez-Ferrer I., Paulus A., Yang Y., Swanberg M., Deierborg T., Boza-Serrano A. (2018). Microglia in Neurological Diseases: A Road Map to Brain-Disease Dependent-Inflammatory Response. Front. Cell. Neurosci..

[30] De Biase L.M., Schuebel K.E., Fusfeld Z.H., Jair K., Hawes I.A., Cimbro R., Zhang H.Y., Liu Q.R., Shen H., Xi Z.X. (2017). Local Cues Establish and Maintain Region-Specific Phenotypes of Basal Ganglia Microglia. Neuron.

[31] Colonna M., Butovsky O. (2017). Microglia Function in the Central Nervous System During Health and Neurodegeneration. Annu. Rev. Immunol..

[32] Rangaraju S., Dammer E.B., Raza S.A., Rathakrishnan P., Xiao H., Gao T., Duong D.M., Pennington M.W., Lah J.J., Seyfried N.T. (2018). Identification and therapeutic modulation of a pro-inflammatory subset of disease-associated-microglia in Alzheimer’s disease. Mol. Neurodegener..

[33] Walker D.G. (2020). Defining activation states of microglia in human brain tissue: An unresolved issue for Alzheimer’s disease. Neuroimmunol. Neuroinflamm..

[34] Crotti A., Ransohoff R.M. (2016). Microglial Physiology and Pathophysiology: Insights from Genome-wide Transcriptional Profiling. Immunity.

[35] Heneka M.T., Carson M.J., El Khoury J., Landreth G.E., Brosseron F., Feinstein D.L., Jacobs A.H., Wyss-Coray T., Vitorica J., Ransohoff R.M. (2015). Neuroinflammation in Alzheimer’s disease. Lancet Neurol..

[36] Liddelow S.A., Barres B.A. (2017). Reactive Astrocytes: Production, Function, and Therapeutic Potential. Immunity.

[37] Oksanen M., Lehtonen S., Jaronen M., Goldsteins G., Hämäläinen R.H., Koistinaho J. (2019). Astrocyte alterations in neurodegenerative pathologies and their modeling in human induced pluripotent stem cell platforms. Cell. Mol. Life Sci..

[38] Augé E., Pelegrí C., Manich G., Cabezón I., Guinovart J.J., Duran J., Vilaplana J. (2018). Astrocytes and neurons produce distinct types of polyglucosan bodies in Lafora disease. Glia.

[39] Pellegrini P., Hervera A., Varea O., Brewer M.K., López-Soldado I., Guitart A., Aguilera M., Prats N., Del Río J.A., Guinovart J.J. (2022). Lack of p62 Impairs Glycogen Aggregation and Exacerbates Pathology in a Mouse Model of Myoclonic Epilepsy of Lafora. Mol. Neurobiol..

[40] Rubio-Villena C., Viana R., Bonet J., Garcia-Gimeno M.A., Casado M., Heredia M., Sanz P. (2018). Astrocytes: New players in progressive myoclonus epilepsy of Lafora type. Hum. Mol. Genet..

[41] Valles-Ortega J., Duran J., Garcia-Rocha M., Bosch C., Saez I., Pujadas L., Serafin A., Cañas X., Soriano E., Delgado-García J.M. (2011). Neurodegeneration and functional impairments associated with glycogen synthase accumulation in a mouse model of Lafora disease. EMBO Mol. Med..

[42] Page M.J., McKenzie J.E., Bossuyt P.M., Boutron I., Hoffmann T.C., Mulrow C.D., Shamseer L., Tetzlaff J.M., Akl E.A., Brennan S.E. (2021). The PRISMA 2020 statement: An updated guideline for reporting systematic reviews. BMJ.

[43] Gumusgoz E., Guisso D.R., Kasiri S., Wu J., Dear M., Verhalen B., Nitschke S., Mitra S., Nitschke F., Minassian B.A. (2021). Targeting Gys1 with AAV-SaCas9 Decreases Pathogenic Polyglucosan Bodies and Neuroinflammation in Adult Polyglucosan Body and Lafora Disease Mouse Models. Neurotherapeutics.

[44] Ahonen S., Nitschke S., Grossman T.R., Kordasiewicz H., Wang P., Zhao X., Guisso D.R., Kasiri S., Nitschke F., Minassian B.A. (2021). Gys1 antisense therapy rescues neuropathological bases of murine Lafora disease. Brain.

[45] Acharya J.N., Satishchandra P., Asha T., Shankar S.K. (1993). Lafora’s disease in south India: A clinical, electrophysiologic, and pathologic study. Epilepsia.

[46] Berthier A., Payá M., García-Cabrero A.M., Ballester M.I., Heredia M., Serratosa J.M., Sánchez M.P., Sanz P. (2016). Pharmacological Interventions to Ameliorate Neuropathological Symptoms in a Mouse Model of Lafora Disease. Mol. Neurobiol..

[47] Sánchez-Elexpuru G., Serratosa J.M., Sanz P., Sánchez M.P. (2017). 4-Phenylbutyric acid and metformin decrease sensitivity to pentylenetetrazol-induced seizures in a malin knockout model of Lafora disease. Neuroreport.

[48] Schwarz G.A., Yanoff M. (1965). Lafora’s disease. Distinct clinico-pathologic form of unverricht’s syndrome. Arch. Neurol..

[49] Chambers J.K., Thongtharb A., Shiga T., Azakami D., Saito M., Sato M., Morozumi M., Nakayama H., Uchida K. (2018). Accumulation of Laforin and Other Related Proteins in Canine Lafora Disease With EPM2B Repeat Expansion. Vet. Pathol..

[50] Menchetti M., Antinori L., Serra G.D., Bertolini G., Rosati M. (2021). Clinical features, imaging characteristics, genetic investigation and histopathologic findings in a Chihuahua dog with Lafora disease. Vet. Rec. Case Rep..

[51] Duran J., Hervera A., Markussen K.H., Varea O., López-Soldado I., Sun R.C., Del Río J.A., Gentry M.S., Guinovart J.J. (2021). Astrocytic glycogen accumulation drives the pathophysiology of neurodegeneration in Lafora disease. Brain.

[52] Muñoz-Ballester C., Berthier A., Viana R., Sanz P. (2016). Homeostasis of the astrocytic glutamate transporter GLT-1 is altered in mouse models of Lafora disease. Biochim. Biophys. Acta.

[53] Muñoz-Ballester C., Santana N., Perez-Jimenez E., Viana R., Artigas F., Sanz P. (2019). In vivo glutamate clearance defects in a mouse model of Lafora disease. Exp. Neurol..

[54] Perez-Jimenez E., Viana R., Muñoz-Ballester C., Vendrell-Tornero C., Moll-Diaz R., Garcia-Gimeno M.A., Sanz P. (2021). Endocytosis of the glutamate transporter 1 is regulated by laforin and malin: Implications in Lafora disease. Glia.

[55] Hegreberg G.A., Padgett G.A. (1976). Inherited progressive epilepsy of the dog with comparisons to Lafora’s disease of man. Fed. Proc..

[56] Israelian L., Nitschke S., Wang P., Zhao X., Perri A.M., Lee J., Verhalen B., Nitschke F., Minassian B.A. (2021). Ppp1r3d deficiency preferentially inhibits neuronal and cardiac Lafora body formation in a mouse model of the fatal epilepsy Lafora disease. J. Neurochem..

[57] Jian Z., Alley M.R., Cayzer J., Swinney G.R. (1990). Lafora’s disease in an epileptic Basset hound. N. Z. Vet. J..

[58] Nitschke S., Chown E.E., Zhao X., Gabrielian S., Petković S., Guisso D.R., Perri A.M., Wang P., Ahonen S., Nitschke F. (2021). An inducible glycogen synthase-1 knockout halts but does not reverse Lafora disease progression in mice. J. Biol. Chem..

[59] Pederson B.A., Turnbull J., Epp J.R., Weaver S.A., Zhao X., Pencea N., Roach P.J., Frankland P.W., Ackerley C.A., Minassian B.A. (2013). Inhibiting glycogen synthesis prevents Lafora disease in a mouse model. Ann. Neurol..

[60] Rai A., Mishra R., Ganesh S. (2017). Suppression of leptin signaling reduces polyglucosan inclusions and seizure susceptibility in a mouse model for Lafora disease. Hum. Mol. Gen..

[61] Sánchez-Elexpuru G., Serratosa J.M., Sánchez M.P. (2017). Sodium selenate treatment improves symptoms and seizure susceptibility in a malin-deficient mouse model of Lafora disease. Epilepsia.

[62] Sinha P., Verma B., Ganesh S. (2021). Trehalose Ameliorates Seizure Susceptibility in Lafora Disease Mouse Models by Suppressing Neuroinflammation and Endoplasmic Reticulum Stress. Mol. Neurobiol..

[63] Varea O., Duran J., Aguilera M., Prats N., Guinovart J.J. (2021). Suppression of glycogen synthesis as a treatment for Lafora disease: Establishing the window of opportunity. Neurobiol. Dis..

[64] Turnbull J., DePaoli-Roach A.A., Zhao X., Cortez M.A., Pencea N., Tiberia E., Piliguian M., Roach P.J., Wang P., Ackerley C.A. (2011). PTG depletion removes Lafora bodies and rescues the fatal epilepsy of Lafora disease. PLoS Genet..

[65] Gumusgoz E., Kasiri S., Guisso D.R., Wu J., Dear M., Verhalen B., Minassian B.A. (2022). AAV-Mediated Artificial miRNA Reduces Pathogenic Polyglucosan Bodies and Neuroinflammation in Adult Polyglucosan Body and Lafora Disease Mouse Models. Neurotherapeutic.

[66] Aso E., Andrés-Benito P., Grau-Escolano J., Caltana L., Brusco A., Sanz P., Ferrer I. (2020). Cannabidiol-Enriched Extract Reduced the Cognitive Impairment but Not the Epileptic Seizures in a Lafora Disease Animal Model. Cannabis Cannabinoid Res..

[67] Bak L.K., Walls A.B., Schousboe A., Waagepetersen H.S. (2018). Astrocytic glycogen metabolism in the healthy and diseased brain. J. Biol. Chem..

[68] Vilchez D., Ros S., Cifuentes D., Pujadas L., Vallès J., García-Fojeda B., Criado-García O., Fernández-Sánchez E., Medraño-Fernández I., Domínguez J. (2007). Mechanism suppressing glycogen synthesis in neurons and its demise in progressive myoclonus epilepsy. Nat. Neurosci..

[69] DiNuzzo M., Mangia S., Maraviglia B., Giove F. (2015). Does abnormal glycogen structure contribute to increased susceptibility to seizures in epilepsy?. Metab. Brain Dis..

[70] Sinadinos C., Valles-Ortega J., Boulan L., Solsona E., Tevy M.F., Marquez M., Duran J., Lopez-Iglesias C., Calbó J., Blasco E. (2014). Neuronal glycogen synthesis contributes to physiological aging. Aging Cell.

[71] Augé E., Cabezón I., Pelegrí C., Vilaplana J. (2017). New perspectives on corpora amylacea in the human brain. Sci. Rep..

[72] Peterson P.K., Toborek M. (2014). Neuroinflammation and Neurodegeneration.

[73] Benusa S.D., George N.M., Dupree J.L. (2020). Microglial heterogeneity: Distinct cell types or differential functional adaptation?. Neuroimmunol. Neuroinflamm..

[74] Deczkowska A., Keren-Shaul H., Weiner A., Colonna M., Schwartz M., Amit I. (2018). Disease-Associated Microglia: A Universal Immune Sensor of Neurodegeneration. Cell.

[75] Keren-Shaul H., Spinrad A., Weiner A., Matcovitch-Natan O., Dvir-Szternfeld R., Ulland T.K., David E., Baruch K., Lara-Astaiso D., Toth B. (2017). A Unique Microglia Type Associated with Restricting Development of Alzheimer’s Disease. Cell.

[76] Song W.M., Colonna M. (2018). The identity and function of microglia in neurodegeneration. Nat. Immunol..

[77] Wang W.Y., Tan M.S., Yu J.T., Tan L. (2015). Role of pro-inflammatory cytokines released from microglia in Alzheimer’s disease. Ann. Transl. Med..

[78] Cherry J.D., Olschowka J.A., O’Banion M.K. (2014). Neuroinflammation and M2 microglia: The good, the bad, and the inflamed. J. Neuroinflamm..

[79] Candlish M., Hefendehl J.K. (2021). Microglia Phenotypes Converge in Aging and Neurodegenerative Disease. Front. Neurol..

[80] Cunningham C., Dunne A., Lopez-Rodriguez A.B. (2019). Astrocytes: Heterogeneous and Dynamic Phenotypes in Neurodegeneration and Innate Immunity. Neuroscientist.

[81] Wiley J.C., Meabon J.S., Frankowski H., Smith E.A., Schecterson L.C., Bothwell M., Ladiges W.C. (2010). Phenylbutyric acid rescues endoplasmic reticulum stress-induced suppression of APP proteolysis and prevents apoptosis in neuronal cells. PLoS ONE.

[82] Ricobaraza A., Cuadrado-Tejedor M., Pérez-Mediavilla A., Frechilla D., Del Río J., García-Osta A. (2009). Phenylbutyrate ameliorates cognitive deficit and reduces tau pathology in an Alzheimer’s disease mouse model. Neuropsychopharmacology.

[83] Ganesh S., Delgado-Escueta A.V., Sakamoto T., Avila M.R., Machado-Salas J., Hoshii Y., Akagi T., Gomi H., Suzuki T., Amano K. (2002). Targeted disruption of the Epm2a gene causes formation of Lafora inclusion bodies, neurodegeneration, ataxia, myoclonus epilepsy and impaired behavioral response in mice. Hum. Mol. Genet..

[84] Duran J., Tevy M.F., Garcia-Rocha M., Calbó J., Milán M., Guinovart J.J. (2012). Deleterious effects of neuronal accumulation of glycogen in flies and mice. EMBO Mol. Med..

[85] Gentry M.S., Dixon J.E., Worby C.A. (2009). Lafora disease: Insights into neurodegeneration from plant metabolism. Trends Biochem. Sci..

[86] Gentry M.S., Guinovart J.J., Minassian B.A., Roach P.J., Serratosa J.M. (2018). Lafora disease offers a unique window into neuronal glycogen metabolism. J. Biol. Chem..

[87] Machado-Salas J., Avila-Costa M.R., Guevara P., Guevara J., Durón R.M., Bai D., Tanaka M., Yamakawa K., Delgado-Escueta A.V. (2012). Ontogeny of Lafora bodies and neurocytoskeleton changes in Laforin-deficient mice. Exp. Neurol..

[88] Luo X.G., Chen S.D. (2012). The changing phenotype of microglia from homeostasis to disease. Transl. Neurodegener..

[89] Stephenson J., Nutma E., van der Valk P., Amor S. (2018). Inflammation in CNS neurodegenerative diseases. Immunology.

[90] Danbolt N.C. (2001). Glutamate uptake. Prog. Neurobiol..

[91] Nwaobi S.E., Cuddapah V.A., Patterson K.C., Randolph A.C., Olsen M.L. (2016). The role of glial-specific Kir4.1 in normal and pathological states of the CNS. Acta Neuropathol..

[92] Amédée T., Robert A., Coles J.A. (1997). Potassium homeostasis and glial energy metabolism. Glia.

[93] Coulter D.A., Eid T. (2012). Astrocytic regulation of glutamate homeostasis in epilepsy. Glia.

[94] DiNuzzo M., Mangia S., Maraviglia B., Giove F. (2014). Physiological bases of the K+ and the glutamate/GABA hypotheses of epilepsy. Epilepsy Res..

[95] Xu J., Song D., Xue Z., Gu L., Hertz L., Peng L. (2013). Requirement of glycogenolysis for uptake of increased extracellular K+ in astrocytes: Potential implications for K+ homeostasis and glycogen usage in brain. Neurochem. Res..

[96] Sanz P., Serratosa J.M. (2020). Neuroinflammation and progressive myoclonus epilepsies: From basic science to therapeutic opportunities. Expert Rev. Mol. Med..

[97] Clark I.A., Vissel B. (2016). Excess cerebral TNF causing glutamate excitotoxicity rationalizes treatment of neurodegenerative diseases and neurogenic pain by anti-TNF agents. J. Neuroinflamm..

[98] Bedner P., Steinhäuser C. (2019). TNFα-Driven Astrocyte Purinergic Signaling during Epileptogenesis. Trends Mol. Med..

[99] Nayak D., Roth T.L., McGavern D.B. (2014). Microglia development and function. Annu. Rev. Immunol..

[100] Ortolano S., Vieitez I., Agis-Balboa R.C., Spuch C. (2014). Loss of GABAergic cortical neurons underlies the neuropathology of Lafora disease. Mol. Brain.

[101] Kwon H.S., Koh S.H. (2020). Neuroinflammation in neurodegenerative disorders: The roles of microglia and astrocytes. Transl. Neurodegener..

[102] Benarroch E.E. (2013). Microglia: Multiple roles in surveillance, circuit shaping, and response to injury. Neurology.

[103] Ben Haim L., Carrillo-de Sauvage M.A., Ceyzériat K., Escartin C. (2015). Elusive roles for reactive astrocytes in neurodegenerative diseases. Front. Cell. Neurosci..

[104] Lull M.E., Block M.L. (2010). Microglial activation and chronic neurodegeneration. Neurotherapeutics.

[105] Romá-Mateo C., Aguado C., García-Giménez J.L., Ibáñez-Cabellos J.S., Seco-Cervera M., Pallardó F.V., Knecht E., Sanz P. (2014). Increased oxidative stress and impaired antioxidant response in Lafora disease. Free Radic. Biol. Med..

[106] Ren M., Guo Q., Guo L., Lenz M., Qian F., Koenen R.R., Xu H., Schilling A.B., Weber C., Ye R.D. (2010). Polymerization of MIP-1 chemokine (CCL3 and CCL4) and clearance of MIP-1 by insulin-degrading enzyme. EMBO Rep..

[107] Lee J.K., Schuchman E.H., Jin H.K., Bae J.S. (2012). Soluble CCL5 derived from bone marrow-derived mesenchymal stem cells and activated by amyloid β ameliorates Alzheimer’s disease in mice by recruiting bone marrow-induced microglia immune responses. Stem Cells.

[108] Szczuciński A., Losy J. (2007). Chemokines and chemokine receptors in multiple sclerosis. Potential targets for new therapies. Acta Neurol. Scand..

[109] Xia M.Q., Qin S.X., Wu L.J., Mackay C.R., Hyman B.T. (1998). Immunohistochemical study of the beta-chemokine receptors CCR3 and CCR5 and their ligands in normal and Alzheimer’s disease brains. Am. J. Pathol..

[110] Rappert A., Biber K., Nolte C., Lipp M., Schubel A., Lu B., Gerard N.P., Gerard C., Boddeke H.W., Kettenmann H. (2002). Secondary lymphoid tissue chemokine (CCL21) activates CXCR3 to trigger a Cl- current and chemotaxis in murine microglia. J. Immunol. Res..

[111] Sui Y., Stehno-Bittel L., Li S., Loganathan R., Dhillon N.K., Pinson D., Nath A., Kolson D., Narayan O., Buch S. (2006). CXCL10-induced cell death in neurons: Role of calcium dysregulation. Eur. J. Neurosci..

[112] Nelson T.E., Gruol D.L. (2004). The chemokine CXCL10 modulates excitatory activity and intracellular calcium signaling in cultured hippocampal neurons. J. Neuroimmunol..

[113] Bi F., Huang C., Tong J., Qiu G., Huang B., Wu Q., Li F., Xu Z., Bowser R., Xia X.G. (2013). Reactive astrocytes secrete lcn2 to promote neuron death. Proc. Natl. Acad. Sci. USA.

[114] Cronk J.C., Filiano A.J., Louveau A., Marin I., Marsh R., Ji E., Goldman D.H., Smirnov I., Geraci N., Acton S. (2018). Peripherally derived macrophages can engraft the brain independent of irradiation and maintain an identity distinct from microglia. J. Exp. Med..

[115] Xu E., Boddu R., Abdelmotilib H.A., Sokratian A., Kelly K., Liu Z., Bryant N., Chandra S., Carlisle S.M., Lefkowitz E.J. (2022). Pathological α-synuclein recruits LRRK2 expressing pro-inflammatory monocytes to the brain. Mol. Neurodegener..

[116] Lyman M., Lloyd D.G., Ji X., Vizcaychipi M.P., Ma D. (2014). Neuroinflammation: The role and consequences. Neurosci. Res..

[117] Liddelow S.A., Guttenplan K.A., Clarke L.E., Bennett F.C., Bohlen C.J., Schirmer L., Bennett M.L., Münch A.E., Chung W.S., Peterson T.C. (2017). Neurotoxic reactive astrocytes are induced by activated microglia. Nature.

[118] Hong S., Beja-Glasser V.F., Nfonoyim B.M., Frouin A., Li S., Ramakrishnan S., Merry K.M., Shi Q., Rosenthal A., Barres B.A. (2016). Complement and microglia mediate early synapse loss in Alzheimer mouse models. Science.

[119] Lian H., Litvinchuk A., Chiang A.C., Aithmitti N., Jankowsky J.L., Zheng H. (2016). Astrocyte-Microglia Cross Talk through Complement Activation Modulates Amyloid Pathology in Mouse Models of Alzheimer’s Disease. J. Neurosci..

[120] Wu T., Dejanovic B., Gandham V.D., Gogineni A., Edmonds R., Schauer S., Srinivasan K., Huntley M.A., Wang Y., Wang T.M. (2019). Complement C3 Is Activated in Human AD Brain and Is Required for Neurodegeneration in Mouse Models of Amyloidosis and Tauopathy. Cell Rep..

[121] Stevens B., Allen N.J., Vazquez L.E., Howell G.R., Christopherson K.S., Nouri N., Micheva K.D., Mehalow A.K., Huberman A.D., Stafford B. (2007). The classical complement cascade mediates CNS synapse elimination. Cell.

[122] Sil S., Ghosh T. (2016). Role of cox-2 mediated neuroinflammation on the neurodegeneration and cognitive impairments in colchicine induced rat model of Alzheimer’s Disease. J. Neuroimmunol..

[123] Markussen K.H., Macedo J., Machío M., Dolce A., Goldberg Y.P., Vander Kooi C.W., Gentry M.S. (2021). The 6th International Lafora Epilepsy Workshop: Advances in the search for a cure. Epilepsy Behav..

[124] Panther E.J., Zelmanovich R., Hernandez J., Dioso E.R., Foster D., Lucke-Wold B. (2022). Ferritin and Neurotoxicity: A Contributor to Deleterious Outcomes for Subarachnoid Hemorrhage. Eur. Neurol..

[125] Sanz P., Garcia-Gimeno M.A. (2020). Reactive Glia Inflammatory Signaling Pathways and Epilepsy. Int. J. Mol. Sci..

[126] Vezzani A., Balosso S., Ravizza T. (2019). Neuroinflammatory pathways as treatment targets and biomarkers in epilepsy. Nat. Rev. Neurol..

[127] Sicca F., Ambrosini E., Marchese M., Sforna L., Servettini I., Valvo G., Brignone M.S., Lanciotti A., Moro F., Grottesi A. (2016). Gain-of-function defects of astrocytic Kir4.1 channels in children with autism spectrum disorders and epilepsy. Sci. Rep..

[128] Aguiar C.C., Almeida A.B., Araújo P.V., de Abreu R.N., Chaves E.M., do Vale O.C., Macêdo D.S., Woods D.J., Fonteles M.M., Vasconcelos S.M. (2012). Oxidative stress and epilepsy: Literature review. Oxidative Med. Cell. Longev..

[129] Terrone G., Balosso S., Pauletti A., Ravizza T., Vezzani A. (2020). Inflammation and reactive oxygen species as disease modifiers in epilepsy. Neuropharmacology.

[130] Ricci G., Volpi L., Pasquali L., Petrozzi L., Siciliano G. (2009). Astrocyte-neuron interactions in neurological disorders. J. Biol. Phys..

[131] Franklin H., Clarke B.E., Patani R. (2021). Astrocytes and microglia in neurodegenerative diseases: Lessons from human in vitro models. Prog. Neurobiol..

[132] Riba M., Del Valle J., Augé E., Vilaplana J., Pelegrí C. (2021). From *Corpora amylacea* to wasteosomes: History and perspectives. Ageing Res. Rev..

[133] Riba M., Augé E., Campo-Sabariz J., Moral-Anter D., Molina-Porcel L., Ximelis T., Ferrer R., Martín-Venegas R., Pelegrí C., Vilaplana J. (2019). *Corpora amylacea* act as containers that remove waste products from the brain. Proc. Natl. Acad. Sci. USA.

[134] Riba M., Augé E., Tena I., Del Valle J., Molina-Porcel L., Ximelis T., Vilaplana J., Pelegrí C. (2021). *Corpora amylacea* in the Human Brain Exhibit Neoepitopes of a Carbohydrate Nature. Front. Immunol..

[135] Jurisch-Yaksi N., Yaksi E., Kizil C. (2020). Radial glia in the zebrafish brain: Functional, structural, and physiological comparison with the mammalian glia. Glia.

[136] Bekris L.M., Khrestian M., Dyne E., Shao Y., Pillai J.A., Rao S.M., Bemiller S.M., Lamb B., Fernandez H.H., Leverenz J.B. (2018). Soluble TREM2 and biomarkers of central and peripheral inflammation in neurodegenerative disease. J. Neuroimmunol..

[137] Carter S.F., Herholz K., Rosa-Neto P., Pellerin L., Nordberg A., Zimmer E.R. (2019). Astrocyte Biomarkers in Alzheimer’s Disease. Trends Mol. Med..

[138] Kwon H.S., Lee E.H., Park H.H., Jin J.H., Choi H., Lee K.Y., Lee Y.J., Lee J.H., de Oliveira F., Kim H.Y. (2020). Early increment of soluble triggering receptor expressed on myeloid cells 2 in plasma might be a predictor of poor outcome after ischemic stroke. J. Clin. Neurosci..

[139] Suárez-Calvet M., Kleinberger G., Araque Caballero M.Á., Brendel M., Rominger A., Alcolea D., Fortea J., Lleó A., Blesa R., Gispert J.D. (2016). sTREM2 cerebrospinal fluid levels are a potential biomarker for microglia activity in early-stage Alzheimer’s disease and associate with neuronal injury markers. EMBO Mol. Med..

[140] Scarf A.M., Kassiou M. (2011). The translocator protein. J. Nucl. Med..

[141] Malpetti M., Kievit R.A., Passamonti L., Jones P.S., Tsvetanov K.A., Rittman T., Mak E., Nicastro N., Bevan-Jones W.R., Su L. (2020). Microglial activation and tau burden predict cognitive decline in Alzheimer’s disease. Brain.

